# Elevating athletic performance: Maximizing strength and power in long jumpers through combined low-intensity blood flow restriction and high-intensity resistance training

**DOI:** 10.1016/j.heliyon.2023.e19068

**Published:** 2023-08-12

**Authors:** Pehzaan Sarfabadi, Moattar Raza Rizvi, Ankita Sharma, Waqas Sami, Mirza Rizwan Sajid, Sumit Arora, Akshay Anand, Mohd Rashid bin Ab Hamid

**Affiliations:** a Department of Physiotherapy, Faculty of Allied Health Sciences, Manav Rachna International Institute and Studies (MRIIRS), Faridabad, 121001, India; b Faculty of Allied Health Sciences, Manav Rachna International Institute and Studies (MRIIRS), Faridabad, 121001, India; c Department of Pre-Clinical Affairs, College of Nursing, QU Health, Qatar University, Doha P.O. Box 2713, Qatar; d Department of Statistics, University of Gujrat, Pakistan; e Manav Rachna Sport Science Centre, Manav Rachna International Institute and Studies (MRIIRS), Faridabad, 121001 India; f Sultan Qaboos Comprehensive Cancer Care and Research Centre, Al Khoud, Muscat, Oman; g Centre for Mathematical Sciences, Universiti Malaysia Pahang Al-Sultan Abdullah, Lebuh Persiaran Tun Khalil Yaakob, 26300, Kuantan, Pahang, Malaysia

**Keywords:** Blood flow restriction therapy, High intensity training, Low resistance training, Isokinetic dynamometer

## Abstract

**Purpose:**

This study aimed to evaluate the effects of low-intensity blood flow restriction (BFR) training and high-intensity resistance training (HI-RT) on the leaping performance of long-jumpers.

**Materials and methods:**

Long jump players were divided into two groups; one group (group A) receiving HI-RT (n = 8) and the other group (group B) receiving combined low-intensity BFR training plus HI-RT (n = 8). Muscle power and knee muscle strength was assessed at baseline, 3 weeks and 6 weeks of intervention.

**Results:**

1-RM was found to be significantly different between Group A and Group B at 3 and 6 weeks. Further, IKDQR, IKDHR and IKDQL was significantly improved in group B as compared to group A both at 3 and 6 weeks. There was significant time effect, group effect and time-group interaction in the strength of quadriceps and hamstring of both left and right leg measured through isokinetic device. Post-hoc analysis for 1-RM in group B showed a significant improvement at baseline and 6 weeks and the broad jump was significant at baseline and 3 weeks and at baseline and 6 weeks.

**Conclusion:**

The combined effects of low-intensity BFR training and HI-RT is effective in improving the muscle strength and power of lower limbs in long jumpers.

## 1 Introduction

The long jump is a crucial part of track and field competition. It was one of the original additions to the Olympic Games. The competition consists of the athlete running along a runway and jumping from a wooden take-off board into a sand pit. Thus, a good long jumper needs to be a fast runner, possess powerful leaping legs, and have good enough coordination to complete the relatively complex take-off, flight, and landing movements. The distance from the leaping point is rather far; therefore, the competitors will need to use all their strength, talent, and effort to make it there[1].

One of the most fundamental ways to boost muscular strength, daily physical function, sports performance, and healing time from orthopedic issues is through resistance training. Traditional high-intensity resistance training (HIRT, 60–80% of one repetition maximum [1RM]) results in the greatest increase in muscular strength, muscle size, and neural adaptability[2]. The high intensity required for muscular adaptation during regular resistance exercise may be impractical and potentially hazardous if performed without proper supervision. It is indicated in many studies that HIRT reduces central arterial compliance in healthy males. Lack of arterial compliance is associated with an increase in systolic blood pressure, the development of coronary heart disease, and a reduction in the sensitivity of the arterial baroreflex[3].

In blood flow restriction (BFR) training, the arterial inflow to a targeted muscle group is reduced while the venous outflow is blocked entirely. As a specialised form of exercise, BFR training involves applying external pressure and using a tourniquet cuff on the upper and lower extremities' proximal joints. When the cuff is inflated, it gradually compacts the veins below it, cutting off arterial blood flow to the distal structures and severely limiting venous outflow, which in turn impedes venous return. Poor oxygen delivery (hypoxia) within the muscle is caused by vessel compression proximal to the skeletal musculature[4,5] Muscle strength and power training in sports have always had as their primary focus the enhancement of the players' abilities in the game's essential physical activities. BFR workouts of low and moderate intensity have been hypothesized to produce adaptations comparable to those of high-intensity resistance training, including increased muscular hypertrophy and strength[6].

Muscle hypertrophy is caused by two main factors, mechanical tension and metabolic stress. Mechanical tension causes increased anabolic hormone levels, which leads to muscle hypertrophy. Metabolic stress causes hormone release, hypoxia, and cell swelling, and these form a part of muscle tissue anabolism[7]. Myogenic stem cells are normally inactive but are activated during increased muscle tension or an injury to a muscle, and they not only help in the repair of damaged muscle fibers but they also help in the growth of muscle fibers[8]. Short-term, low-intensity BFR training for 4–6 weeks has been found to increase muscle strength by 10–20%. These surges were comparable to those gained from high-intensity exercise without BFR[9]. By using a cuff to replicate a hypoxic environment, BFR training aims to mimic the consequences of intense exercise. After positioning the cuff proximally to the muscle being exercised, light exercises can be performed. The accumulation of low-oxygen blood causes a rise in proton and lactic acid because of the cuff's restriction of blood flow[10]. BFR training and low-intensity exercise cause the same physiological changes in the muscles as high-intensity exercise, including hormone release, hypoxia, and cell swelling[11].

There is a dearth of research on the combined effects of low-intensity BFR training and HIRT on athletic performance, despite the plethora of studies on the benefits of both training modalities taken individually. The goal of this research was to determine if long jumpers could improve their performance by combining low-intensity BFR training with HIRT.

## 2 Materials and Methods

### 2.1 Sample size

The study's sample size was determined using statistical software, G*Power version 3.1.9.4 (Universität Kiel, Kiel, Germany). According to an a priori power analysis for a repeated-measures analysis of variance that examined main effects and interactions with two groups and three repeated-measures, the sample size was found to be 28 in order to detect a medium effect (η^2^p = 0.05) assuming 80% power in our dependent measures of interest.

### 2.2 Study design

The participants were recruited following the convenience sampling method, which involves choosing individuals who are readily available and accessible. A randomised experimental design was used to investigate the combined effect of low-intensity blood flow restriction training combined with high-intensity resistance training. Randomization was performed using a double-blind experiment to eliminate bias, where neither the participants nor the evaluators were aware of the group assignments. Further to assign participants to different treatment groups, a balanced randomization procedure was utilized. This process involved the use of sealed opaque envelopes containing group assignment numbers, which were generated randomly by a computer.

### 2.3 Inclusion and exclusion criteria

The long jumpers participating at the district level matches and engaged for more than 6 months to 2 years and practicing at least 3–4 times a week and above were included in this study. Athletes whose competitive season had ended and who were not scheduled to participate in any competition within the following eight weeks were included in the study. Long jumpers having resting blood pressure greater than 90 mmHg and 140 mmHg, having any orthopedic, or neurological or cardiovascular diseases, or currently taking any medication, having a history of increased blood clotting factor, or being smokers were excluded.

**Fig. 1:**
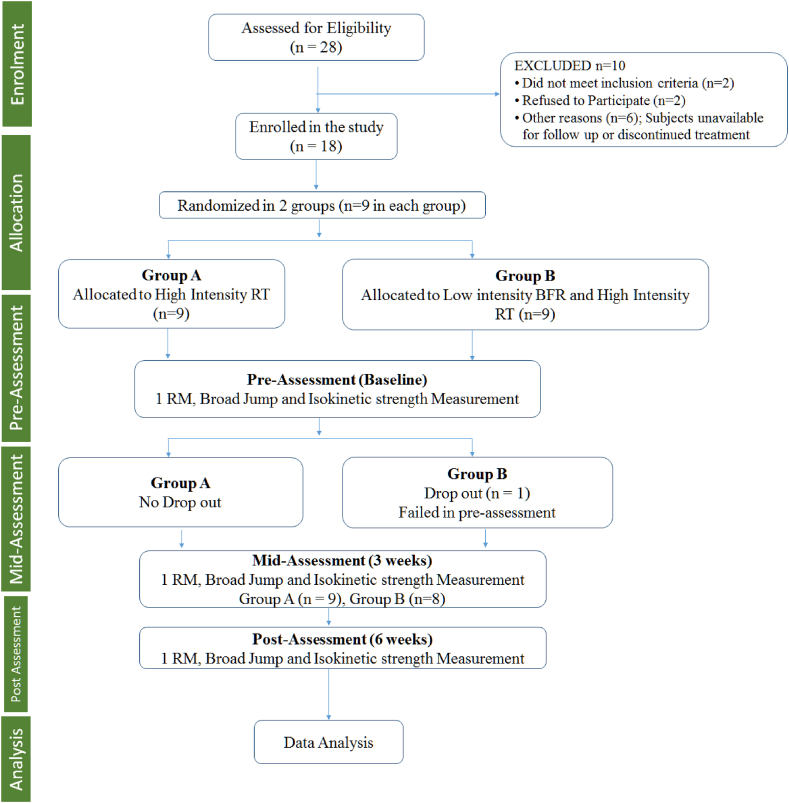
The CONSORT (Consolidated Standards of Reporting Trials) flow diagram demonstrating the detailed information of sampling and interventions received.

Twenty-eight long jumpers were screened based on exclusion and inclusion criteria. The pre-screening was done before performing BFR training, and it involved measurements of blood pressure, temperature, resting heart rate, resting respiratory rate, and Homan's sign (to check for Deep vein thrombosis (DVT)). Ten long jumpers were excluded for not meeting the inclusion criteria (n = 2), denying to participate (n = 2) and other reasons (n = 6). The study included eighteen long jumpers who were randomly assigned to group A (n = 9, HIRT) and group B (n = 9, Low intensity BFR + HIRT). A pre-assessment was done for one repetition maximum (1-RM), broad jump and isokinetic testing to evaluate the strength of the quadriceps and hamstring muscles in the left and right leg. One long jumper in the group B was dropped after failing to perform while on BFR training. Therefore, nine long jumpers in group A and eight long jumpers in group B continued for mid assessment of the same outcome measures at 3 weeks, followed by post assessment at 6 weeks (Fig. 1).

### 2.4 One repetition maximum (1-RM) strength measurement

For the measurement of one repetition maximum (1-RM), the participants were asked for the predicted weight for by which they can complete only one squat for full ROM [12] They completed controlled leg squats with predicted weight of one set of 5 repetitions at 50% of predicted weight of 1-RM, 3 repetitions at 60%, 2 repetitions at 80%, and then 1 repetition at 90%, and 1 repetition at 95%, 1 repetition at 100% 5–7 trials were required to complete the test. The athlete completed the 100% lift successfully, the weight was increased in 2.5 kg increments until failure. Between attempts, athletes were given a 5 min of recovery period. Athletes made three maximal efforts on average to ascertain their genuine 1-RM[13]. The familiarization trial 1-RM, for the device, how to lift weight and meaning of complete range of motion was given a day prior to the assessment.

### 2.5 Broad jump test

The traditional warm-up consisted of 5 min of jogging and 5 min of active stretching. Each participant positioned his or her legs parallel and feet shoulder-width apart at the starting line. Participants were told to bend their knees to a self-selected degree and bring their arms behind their bodies. They then extended their legs, swung their arms forward, and jumped as far as possible with a tremendous drive. The distance jumped was measured in centimetres[14].

### 2.6 Maximum isokinetic strength measurement

The isokinetic strength was measured using the TecnoBody IsoMove (IND IsoDyno) isokinetic dynamometer, employing the previously utilized technique [15,16]. The isokinetic dynamometer allows for isokinetic contraction at a variety of predefined velocities. The isokinetic dynamometer offers resistance by matching the force applied to it and preventing acceleration above the pre-set velocity of movement. Once the participant was secured in the chair, the range-of-motion limits were calculated and set using goniometry. The maximum isometric torque (endpoint) was measured at a velocity of zero degrees of full knee extension, with the lever arm locked in a position of 90° knee flexion. The angular velocity was kept at 240°/s for knee flexion and extension while measurement[17]. To decrease the possibility of erroneous data, gravity adjustment was conducted on each leg prior to testing. After making sure the individual was seated and secured, they performed three continuous repetitions for three sets with a rest time of 10 s between each set. The individuals were told to perform all activities as quickly and forcefully as possible in order to achieve maximum torque[18].

### 2.7 Training protocols

Group A performed high-intensity resistance training, while group B performed a combination of low-intensity BFR training and high-intensity resistance training. The exercises performed as HI-RT were same for both the groups. The LI-BFR training load was stable throughout the entire cycle. To rule out any dietary variation in measuring muscular strength and size, participants were instructed to stick to the same diets during the training session. They were also instructed to abstain from consuming coffee and alcohol for the full day leading up to the pre- and post-training assessments.

High Intensity Resistance Training (HI-RT) was administered in accordance with the guidelines established in the previous study[19]. The guidelines recommend conducting HI-RT sessions twice per week for a duration of 32 min per session. The recommended intensity is 6–8 maximal repetitions (RM), and 1–3 sets were given for leg press and weighted squats at 60–70% of 1 RM. Individuals aimed for six to eight repetitions, achieving as many repetitions as they can within 60 s. Active recovery at low intensity, such as keeping a heart rate reserve of 30–40%, is highly suggested over passive recovery. Maximum heart rate was calculated using formula 220-age[20]. Following these rules can assist individuals in safely and efficiently engaging in HI-RT to reach their fitness goals.

Patterson et al. [21] developed guidelines for BFR training to optimize its effectiveness[21]. These recommendations suggest doing BFR training twice weekly for six weeks. The maximum time was restricted to 5–10 min per exercise, with rest time in between the sets, and the training range should be 20% of the one-repetition maximum (1 RM). Recovery periods should be between 20 and 30 s between exercises and between 2 and 3 min between the sets. Four sets of BFR exercises were performed, with the leg press exercise and weighted squats focusing on quadriceps and hamstrings. The cuff size ought to be determined by the person's limb circumference and the width of the cuff approximately 10 cms. A total of 75 repetitions are advised for BFR training, with 30 warm-up repeats without BFR, three sets of 15 BFR repetitions, or sets to failure. The suggested occlusion pressure for BFR training is 60% of the femoral artery's arterial occlusion pressure. The specific pressure used for BFR training was calculated by multiplying the individual's femoral artery occlusion pressure by 0.6. The base reference value for each individual's femoral artery occlusion pressure was obtained through a standardized measurement procedure. This procedure typically involves using a specialised Doppler ultrasound device to measure the occlusion pressure, which is the minimum pressure required to completely occlude blood flow in the femoral artery. This reference value serves as a baseline measurement and is unique to each individual. To determine the pressure for BFR training, 60% of the individual's femoral artery occlusion pressure was calculated, providing a targeted pressure that is customized to the individual's physiological characteristics and occlusion threshold. This personalized approach helps optimize the effectiveness and safety of blood flow restriction training for each participant.

The recovery period within sets ought to range from 30 to 60 s, and the exercise should be performed with continuous limitation. For the concentric and eccentric phases of BFR exercises, the suggested execution speed is 1–2 s. The exercise should be repeated until concentric failure or the desired repetition scheme is reached.

### 2.8 Statistical analyses

All analyses were performed using Statistical Package for the Social Sciences (v25.0, IBM Corporation, New York, USA). The normal distribution of data was assessed using the Shapiro-Wilks test. Descriptive statistics (mean ± SD) summarized the data, while the independent *t*-test compared baseline characteristics of two independent groups. To check the homogeneity of variances across groups, Levene's test was conducted for each variable. A two-way ANOVA with repeated measures examined the effects of two independent factors on a dependent variable. Mauchly's test of sphericity was used to verify the assumption of sphericity for the two-way ANOVA. The level of statistical significance was set at p ≤ 0.05. Effect sizes were reported using Partial Eta-squared (η^2^) and Cohen's d. Partial Eta-squared provided an estimate of the proportion of variance in the dependent variable accounted for by each factor and their interactions, while Cohen's d quantified the standardized mean difference between groups or conditions. Effect sizes of 0.2, 0.5, and 0.8 were interpreted as small, medium, and large, respectively.

## 3 Results

The average weight for Group A (baseline: 65.45 ± 3.42 Kg; 3 weeks: 65.3 ± 24.51 Kg; 6 weeks: 65.1 ± 74.54 Kg, p = 0.67) and Group B (baseline: 64.36 ± 5.52 Kg; 3 weeks: 65.07 ± 4.96 Kg; 6 weeks: 65.72 ± 4.68 Kg, p = 0.79) remained consistent throughout the duration of the 6-week research. Similarly the average height of both Group A (Baseline: 161.58 ± 5.69 cm; 3 weeks: 164.37 ± 5.84 cm; 6 weeks: 162.34 ± 6.04 cm, p = 0.10) and the Group B (Baseline: 162.71 ± 7.83 cm; 3 weeks: 163.28 ± 6.98 cm; 6 weeks: 162.89 ± 6.79, p = 0.18) remained the same throughout the study period.

**Fig. 2:**
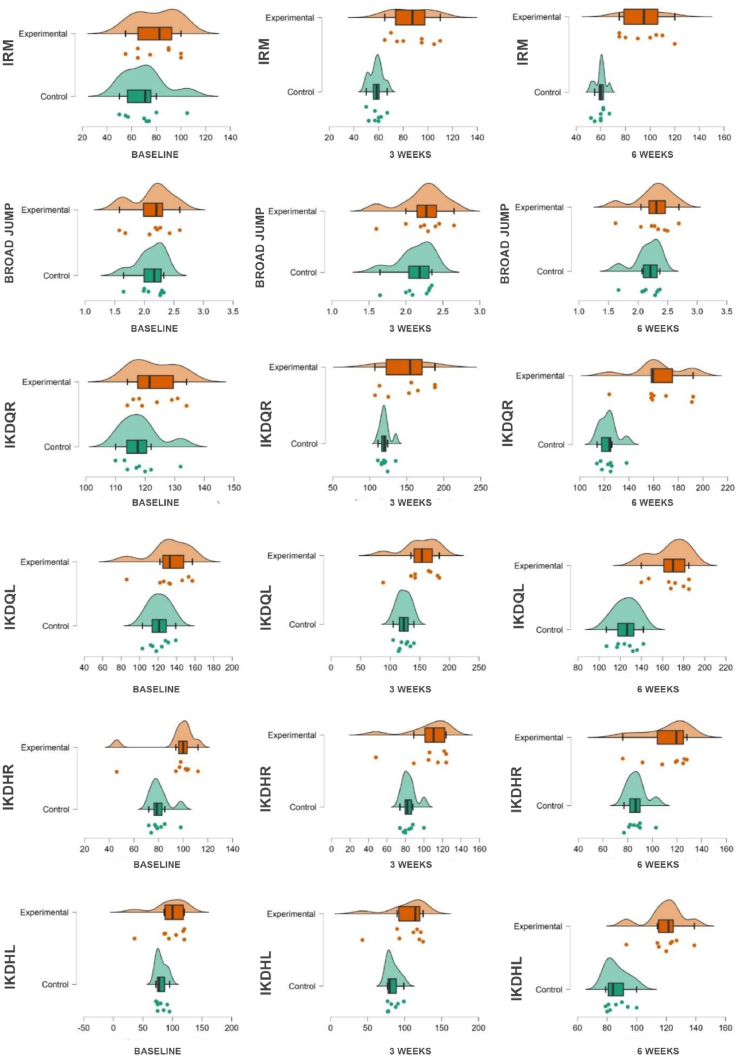
A raincloud plot is shown, which depicts the distribution of outcome variables for two groups (Groups A and B) over three time points (baseline, 3 weeks, and 6 weeks). A kernel density curve and box plots for each group and time point are included in the plot. 1-RM- (1 Repetition Maximum) measured in kilogram, BJ- Broad Jump in meters and IKDQR (Isokinetic Device Quadriceps Right), IKDHR (Isokinetic Device Hamstring Right), IKDQL (Isokinetic Device Quadriceps Left) and IKDHL (Isokinetic Device Hamstring Left) measure in newton-meters.

Independent sample *t*-test showed that there was no significant difference in the baseline scores between Group A and Group B in all the outcome variables (Fig. 2). 1-RM was found to be significantly different between Group A and Group B both at 3 (p < 0.001) and 6 weeks (p < 0.001). The effect size (Cohen's d) for both time points was large, indicating a substantial difference between the groups. There was no significant difference in broad jump between Group A and B at baseline (p = 0.89), 3 weeks (p = 0.49) and 6 weeks (p = 0.45). The effect size was small for all time points, suggesting that the difference between the groups was not substantial. Further, IKDQR (Isokinetic Device Quadriceps Right), IKDHR (Isokinetic Device Hamstring Right) and IKDQL (Isokinetic Device Quadriceps Left) were significantly higher in group B as compared to group A both at 3 and 6 weeks. IKDHL (Isokinetic Device Hamstring Left) was found to be significantly greater in group B only at 6 weeks but not at 3 weeks (Table 1).

**Table 1:** Comparison of muscle strength and power in group A and group B at baseline (pre-assessment), 3 weeks (mid-assessment) and 6 weeks (post-assessment) using independent sample *t*-test.

Outcome variable	Time	Mean ± Std. Deviation		t	p	Cohen's d	95% CI	
		Group A	Group B				Lower	Upper
1-RM	Baseline	70.38 ± 17.48	80.0 ± 17.32	−1.11	0.29	−0.55	−28.28	9.03
	3 weeks	58.13 ± 5.44	86.88 ± 16.68	−4.64	**p<0.001**	−2.32	−42.05	−15.45
	6 weeks	59.75 ± 4.56	94.38 ± 17.00	−5.57	**p<0.001**	−2.78	−47.97	−21.28
BJ	Baseline	2.11 ± 0.23	2.13 ± 0.35	−0.14	0.89	−0.07	−0.34	0.30
	3 weeks	2.13 ± 0.24	2.23 ± 0.32	−0.71	0.49	−0.36	−0.40	0.20
	6 weeks	2.16 ± 0.23	2.27 ± 0.33	−0.77	0.45	−0.39	−0.41	0.19
IKDQR	Baseline	118.25 ± 6.78	123.13 ± 7.49	−1.36	0.19	−0.68	−12.54	2.79
	3 weeks	120.25 ± 7.13	149.38 ± 31.62	−2.54	**0.02**	−1.27	−53.70	−4.55
	6 weeks	123.25 ± 7.40	164 ± 21.59	−5.05	**p<0.001**	−2.53	−58.05	−23.45
IKDQL	Baseline	121.13 ± 11.62	131.88 ± 22.41	−1.20	0.25	−0.60	−29.89	8.39
	3 weeks	123.0 ± 11.54	150.38 ± 31.02	−2.34	**0.03**	−1.17	−52.47	−2.28
	6weeks	125.63 ± 11.40	167.88 ± 16.76	−5.89	**p<0.001**	−2.95	−57.62	−26.88
IKDHR	Baseline	80.63 ± 8.14	94.50 ± 20.34	−1.79	0.09	−0.90	−30.49	2.74
	3 weeks	83.75 ± 7.96	104.13 ± 25.67	−2.14	**0.05**	−1.07	−40.75	0.00
	6weeks	87 ± 7.92	111.75 ± 18.69	−3.45	**p<0.001**	−1.72	−40.15	−9.35
IKDHL	Baseline	80.88 ± 8.58	96.0 ± 28.04	−1.46	0.17	−0.73	−37.36	7.11
	3 weeks	83.75 ± 8.14	102.75 ± 27.46	−1.88	0.10	−0.94	−40.72	2.72
	6weeks	86.50 ± 7.60	119.38 ± 13.21	−6.11	**p<0.001**	−3.05	−44.42	−21.33

1-RM- 1 Repetition Maximum, BJ- Broad Jump, IKDQR- Isokinetic Device Quadriceps Right, IKDHR- Isokinetic Device Hamstring Right, IKDQL- Isokinetic Device Quadriceps Left, IKDHL- Isokinetic Device Hamstring Left.

**Fig. 3:**
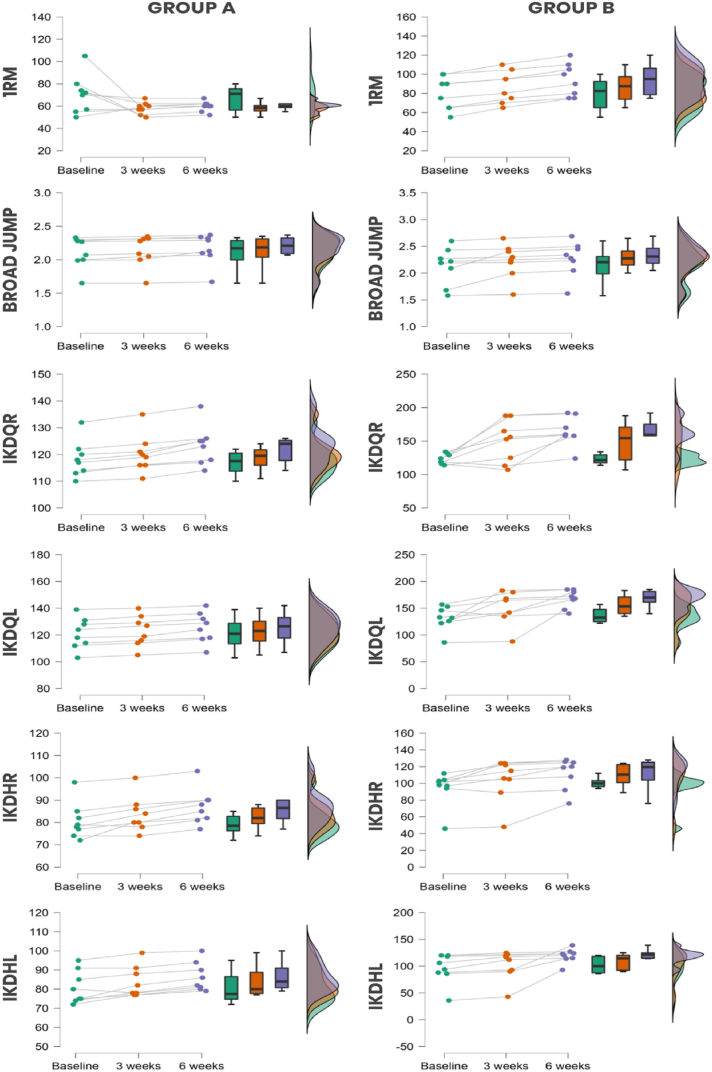
The line, box, and density plots from repeated measure ANOVA in this raincloud display show the distribution of scores for individual participants in Group A and Group B at three time points (baseline, 3 weeks, and 6 weeks). 1-RM- (1 Repetition Maximum) measured in kilogram, BJ- Broad Jump in meters and IKDQR (Isokinetic Device Quadriceps Right), IKDHR (Isokinetic Device Hamstring Right), IKDQL (Isokinetic Device Quadriceps Left) and IKDHL (Isokinetic Device Hamstring Left) measure in newton-meters.

For 1-RM, repeated measure ANOVA showed no time effect (p = 0.27) but there was significant group effect (p < 0.001) and the time-group interaction (p = 0.002) was also significant (Fig. 3). Broad Jump results showed that there was significant time effect (p < 0.001) however there was no significant difference in group effect (p = 0.09) and time-group interaction (p = 0.60). There was significant time effect, group effect and time-group interaction in the strength of quadriceps and hamstring of both left and right leg measured through isokinetic device (Table 2).

**Table 2:** Repeated-measures ANOVA of outcome variables (muscle strength and power) to assess changes over time at baseline (pre-assessment), 3 weeks (mid-assessment) and 6 weeks (post-assessment).

Outcome variables	Baseline		3 Weeks		6 weeks		Time Effect		Group Effect		Time × group interaction	
	Group A	Group B	Group A	Group B	Group A	Group B	p	pη^2^	p	pη^2^	p	pη2
1-RM	70.38 ± 17.48	80.0 ± 17.32	58.13 ± 5.44	86.88 ± 16.68	59.75 ± 4.56	94.38 ± 17.00	0.27	0.09	**0.002**	0.51	**<0.001**	0.44
BJ	2.11 ± 0.23	2.13 ± 0.35	2.13 ± 0.24	2.23 ± 0.32	2.16 ± 0.23	2.27 ± 0.33	**<0.001**	0.43	0.60	0.02	0.09	0.16
IKDQR	118.25 ± 6.77	123.13 ± 7.50	120.25 ± 7.13	149.38 ± 31.62	123.25 ± 7.40	164 ± 21.59	**<0.001**	0.58	**0.004**	0.47	**<0.001**	0.46
IKDHR	80.63 ± 8.14	94.50 ± 20.34	83.75 ± 7.96	104.13 ± 25.67	87 ± 7.92	111.75 ± 18.69	**<0.001**	0.60	**0.03**	0.31	**0.02**	0.24
IKDQL	121.13 ± 11.62	131.88 ± 22.41	123.0 ± 11.54	150.38 ± 31.02	125.63 ± 11.40	167.88 ± 16.76	**<0.001**	0.51	**0.007**	042	**0.001**	0.39
IKDHL	80.88 ± 8.58	96.0 ± 28.04	83.75 ± 8.14	102.75 ± 27.46	86.50 ± 7.60	119.38 ± 13.21	**<0.001**	0.45	**0.02**	0.35	**0.02**	0.25

1-RM- 1 Repetition Maximum, BJ- Broad Jump, IKDQR- Isokinetic Device Quadriceps Right; IKDHR- Isokinetic Device Hamstring Right, IKDQL- Isokinetic Device Quadriceps Left, IKDHL- Isokinetic Device Hamstring Left.; *(p < 0.05) value of significance.

In terms of post hoc test for 1RM, Group A showed a significant decrease at 3 weeks and 6 weeks compared to baseline, while Group B did not show any significant changes compared to baseline. Group B did, however, show a significant increase in 1RM at 6 weeks compared to baseline. For BJ, Group A did not show any significant changes compared to baseline, while Group B showed a significant increase at both 3 weeks and 6 weeks. There were no significant differences in 1RM and BJ between Group A and Group B at any time point (Table 3).

**Table 3:** Posthoc (Bonferroni) Pairwise comparison (Time effect) of power between group A and group B at baseline (pre-assessment), 3 weeks (mid-assessment) and 6 weeks (post-assessment).

Outcome Variables	Group	Group	MD	t	pbonf
1-RM	Group A, Baseline	Group B, Baseline	−9.63	−1.35	1.00
	Group A, Baseline	Group A, 3 weeks	12.25	3.13	0.06
	Group A, Baseline	Group A, 6 weeks	10.63	2.71	0.17
	Group B, Baseline	Group B, 3 weeks	−6.88	−1.76	1.00
	Group B, Baseline	Group B, 6 weeks	−14.38	−3.67	0.02*
	Group A, 3 weeks	Group A, 6 weeks	−1.63	−0.42	1.00
	Group B, 3 weeks	Group B, 6 weeks	−7.50	−1.92	0.98
BJ	Group A, Baseline	Group B, Baseline	−0.02	−0.15	1.00
	Group A, Baseline	Group A, 3 weeks	−0.02	−0.68	1.00
	Group A, Baseline	Group A, 6 weeks	−0.05	−1.70	1.00
	Group B, Baseline	Group B, 3 weeks	−0.10	−3.35	0.04*
	Group B, Baseline	Group B, 6 weeks	−0.14	−4.67	0.001**
	Group A, 3 weeks	Group A, 6 weeks	−0.03	−1.02	1.00
	Group B, 3 weeks	Group B, 6 weeks	−0.04	−1.32	1.00
IKDQR	Group A, Baseline	Group B, Baseline	−4.88	−0.58	1.00
	Group A, Baseline	Group A, 3 weeks	−2.00	−0.38	1.00
	Group A, Baseline	Group A, 6 weeks	−5.00	−0.94	1.00
	Group B, Baseline	Group B, 3 weeks	−26.25	−4.94	<0.001***
	Group B, Baseline	Group B, 6 weeks	−40.88	−7.70	<0.001***
	Group A, 3 weeks	Group A, 6 weeks	−3.00	−0.57	1.00
	Group B, 3 weeks	Group B, 6 weeks	−14.63	−2.75	0.15
IKDQL	Group A, Baseline	Group B, Baseline	−10.75	−1.14	1.00
	Group A, Baseline	Group A, 3 weeks	−1.88	−0.35	1.00
	Group A, Baseline	Group A, 6 weeks	−4.50	−0.85	1.00
	Group B, Baseline	Group B, 3 weeks	−18.50	−3.48	0.03*
	Group B, Baseline	Group B, 6 weeks	−36.00	−6.76	<0.001***
	Group A, 3 weeks	Group A, 6 weeks	−2.63	−0.49	1.00
	Group B, 3 weeks	Group B, 6 weeks	−17.50	−3.29	0.04*
IKDHR	Group A, Baseline	Group B, Baseline	−13.88	−1.69	1.00
	Group A, Baseline	Group A, 3 weeks	−3.13	−1.20	1.00
	Group A, Baseline	Group A, 6 weeks	−6.38	−2.44	0.32
	Group B, Baseline	Group B, 3 weeks	−9.63	−3.69	0.01*
	Group B, Baseline	Group B, 6 weeks	−17.25	−6.61	<0.001***
	Group A, 3 weeks	Group A, 6 weeks	−3.25	−1.25	1.00
	Group B, 3 weeks	Group B, 6 weeks	−7.63	−2.92	0.10
IKDHL	Group A, Baseline	Group B, Baseline	−15.13	−1.70	1.00
	Group A, Baseline	Group A, 3 weeks	−2.88	−0.66	1.00
	Group A, Baseline	Group A, 6 weeks	−5.63	−1.29	1.00
	Group B, Baseline	Group B, 3 weeks	−6.75	−1.55	1.00
	Group B, Baseline	Group B, 6 weeks	−23.38	−5.38	<0.001***
	Group A, 3 weeks	Group A, 6 weeks	−2.75	−0.63	1.00
	Group B, 3 weeks	Group B, 6 weeks	−16.63	−3.83	0.01*

1-RM- 1 Repetition Maximum; BJ- Broad Jump; MD, Mean difference; IKDQR- Isokinetic Device Quadriceps Right, IKDHR- Isokinetic Device Hamstring Right, IKDQL- Isokinetic Device Quadriceps Left, IKDHL- Isokinetic Device Hamstring Left; *p < 0.05, **p < 0.01, ***p < 0.001.

Specifically, Group B showed a significant increase in IKDQR and IKDQL at 3 weeks and 6 weeks compared to baseline, while Group A did not show any significant changes at any time point. Further, there were significant differences in IKDQR and IKDQL between Group B at 3 weeks and 6 weeks compared to Group B at baseline, indicating that the intervention had a significant effect on this group. The post hoc analysis of the IKDHR and IKDHL data showed that there were no significant differences between Group A and Group B at baseline for either measure. For IKDHR, there were significant increase for Group B at 3 weeks and 6 weeks compared to baseline, while there were no significant changes for Group A. In addition, Group B showed significant difference in IKDQL between 3 weeks and 6 weeks. For IKDHL, there were no significant changes for Group A at any time point, but Group B showed a significant increase at 3 weeks and 6 weeks compared to baseline. There were no significant differences between any of the time points for Group A for IKDHR, and a significant trend for an increase in IKDQR at 3 weeks compared to 6 weeks for Group B (Table 3).

## 4 Discussion

As a specialised method, blood-flow-restriction (BFR) training uses a pneumatic compression device to apply external pressure and a tourniquet cuff to the proximal areas of the upper and lower extremities. It is important for long jumpers to have strong, powerful legs to increase their athletic potential and avoid the kinds of injuries that are common in their sport. The study set out to compare the effectiveness of low-intensity BFR training with high-intensity resistance training (HI-RT) in improving long-jumpers' explosiveness and strength. According to the results of the current study, long-jumpers engaged in low-intensity BFR training in combination with HI-RT observed a significant increase in muscle strength. After 6 weeks, the athletes had improved their long jump performances, which they attributed to a noticeable increase in broad jump and 1-RM in both their quadriceps and hamstrings. The results of a previous comparable study[22] indicated that combining HI-RT and low-intensity BFR training produced better results than either training alone.

The combination of low-intensity BFR training with HI-RT resulted in increases in isometric and dynamic strength as well as isokinetic strength. Combining HI-RT with low-intensity BFR has been demonstrated to promote muscle size and strength in previous studies[[23], [24], [25]]. One of the most important physiological characteristics of BFR training is the release of growth hormone[5]. It has also been hypothesized that when people engage in BFR training, their levels of systemic growth hormone rise. Resistance exercise combined with BFR training activates circulating substances like growth hormone, and endocrine system, leading to muscular hypertrophy[13,24]. Low-intensity training with BFR increased the quadriceps and hamstring strength of long jumpers. Low-intensity BFR elicited the same muscle adaptations as HI-RT. This has been brought to light by previous studies on HI-RT[23,26] which also reveal similar volumes of increases in muscle size and isometric and 1 repetition maximum (1-RM) strength. HI-RT has been reported to increase muscle protein synthesis in a single bout of exercise[27]. Similar results were observed with low-intensity BFR training[28].

1-RM was considered as an indicator of the strength. There was a significant increase in 1-RM squat performance in group B receiving a combination of low-intensity BFR training and HI-RT as compared to group A receiving HI-RT in the present study. There was a significant increase in 1-RM in similar studies [11,29] that were performed with BFR training [22]. also found that muscular strength when measured by 1-RM improved in the group B where the low intensity resistance training was combined with BFR as compared to the group A receiving HI-RT.

Compared to the baseline assessment, both strength and power significantly improved following low intensity BFR combined with HI-RT both at 3 weeks and at 6 weeks[30]. However, the results were more remarkable at 6 weeks when compared to 3 weeks. In a previous study that took place over 3 weeks, trained athletes showed that their muscle strength and power increased during that time[31]. Few studies using BFR training and resistance exercises for 4 weeks have also shown remarkable results[13].

Compared to the combined effects of HI-RT and low-intensity BFR training on muscle adaptations, low-intensity BFR training alone has not been able to influence a relative change in isometric and dynamic strength, according to previous studies [22,23,28]. In the current study, the combination of low-intensity BFR training and HI-RT resulted in a significant increase in muscle strength and power. A previous study performed with low-intensity BFR training and HI-RT marked an increase in dynamic strength, but there was no increase in isometric strength[32].

HI-RT in contrast, had a positive influence on both dynamic and isokinetic strength. The frequency of training sessions in both groups A and B kept same in this study, which could have influenced the relative strength outcomes. Group B received two session per week of combination of a low intensity BFR and 3 sessions of HI-RT, while group A received HI-RT three sessions per week[33]. The HI-RT required for muscle adaptation with conventional resistance training may be impractical or even dangerous if performed without competent supervision. Several studies published in the last decade have shown that low-intensity resistance training (20–30% 1-RM) combined with BFR can result in muscle growth [22,28]. The muscle strength and growth improves due to the low mechanical stress and limited muscle damage associated with BFR training, it does not necessitate a considerable recuperation period between sessions[23,29,34].

It was also found that there is a strong correlation between 1-RM squats and standing broad jumps (SBJ)[35]. The present study demonstrated a significant increase in SBJ in the long jump athletes after 3 and 6 weeks. According to another study, it was found that unlike HI-RT, BFR training enhances muscle size and carotid arterial compliance [7]. As a result, BFR training could be a good strategy for boosting muscle growth while reducing the risk of injury. There were few limitations in this study but the major concern was that the physiological mechanism of change in strength and power was not studied. Moreover, the improvement in the muscle girth and the recruitment of muscle fibres was not taken into account.

## 5 Conclusion

Combining low-intensity blood flow restriction training with high-intensity resistance training improves the performance of long jumpers, according to the study. This combination also results in a greater increase in strength than high-intensity resistance training alone. Future research should investigate the effects of this combination of exercises on the core muscles and emphasize on the training of individual muscles.

### 5.1 Limitations of the study

Small sample size, brief study duration, and the absence of double-blinded randomization limit the generalizability of findings from combined BFR and resistance training studies. Performance measures, such as strength and power outcomes, may vary between tests, making it difficult to compare the results. In the study design and analysis, participant characteristics such as initial fitness levels were not considered.

## Production notes

### Author contribution statement

Pehzaan Safarbadi and Ankita Sharma: Conceived and designed the experiments; Performed the experiments; Wrote the paper.

Moattar Raza Rizvi: Conceived and designed the experiments; Analyzed and interpreted the data.

Waqas Sami: Designed the methodology,Analyzed,interpreted the data; Contributed reagents, materials, analysis tools or data; Wrote the paper

Mirza Rizwan Sajid: Conceived and designed the experiments; Wrote the paper.

Sumit Arora and Akshay Anand: Performed the experiments; Wrote the paper.

Mohd Rashid Ab Hamid: Contributed reagents, materials, analysis tools or data; Wrote the paper.

### Data availability statement

Data will be made available on request.

### Funding

Qatar National Library funded the publication of this article.

## Ethical approval

Ethical approval was obtained from the Ethical committee at Faculty of Allied Health Science in accordance to ethical principles for medical research involving human (WMA declaration of Helsinki) having reference no: MRIIRS/FAHS/DEC/2021-23 dated 10th February 2022.

## Declaration of competing interest

The authors declare that they have no known competing financial interests or personal relationships that could have appeared to influence the work reported in this paper.
